# Skipping breakfast, poor sleep quality, and Internet usage and their relation with unhappiness in Japanese adolescents

**DOI:** 10.1371/journal.pone.0235252

**Published:** 2020-07-27

**Authors:** Yuichiro Otsuka, Yoshitaka Kaneita, Osamu Itani, Maki Jike, Yoneatsu Osaki, Susumu Higuchi, Hideyuki Kanda, Aya Kinjo, Yuki Kuwabara, Hisashi Yoshimoto

**Affiliations:** 1 Division of Public Health, Department of Social Medicine, Nihon University School of Medicine, 30–1 Oyaguchi-kamimachi, Itabasi-ku, Tokyo, Japan; 2 Division of Environmental and Preventive Medicine, Department of Social Medicine, Faculty of Medicine, Tottori University, Yonago-city, Tottori, Japan; 3 National Hospital Organization Kurihama Medical and Addiction Center, Yokosuka-city, Kanagawa, Japan; 4 Department of Public Health, Shimane University Faculty of Medicine, Matsue-city, Shimane, Japan; 5 Department of Family Medicine, General Practice and Community Health, Faculty of Medicine, University of Tsukuba, Tsukuba-city, Ibaraki, Japan; Iwate Medical University, JAPAN

## Abstract

Subjective happiness is often regarded as a major life goal. Although Japan is an economically powerful country, the level of subjective well-being reported among Japanese adolescents is lower than in other countries. We aimed to investigate the lifestyle factors related to unhappiness in Japanese adolescents. We collected data through the 2017–2018 Lifestyle Survey of Adolescents, a nationally representative cross-sectional study enrolled in randomly selected junior and senior high schools throughout Japan. We assessed the prevalence of subjective unhappiness in junior and senior high school students according to school life factors and daily lifestyle habits. A multivariable logistic regression analysis was used to examine the associations between these factors and unhappiness. A total of 64,329 students were included in the sample (mean age 15.7 years, 53.9% boys). The average prevalence of unhappiness was 10.2%. The logistic regression analyses indicated that unhappiness was strongly associated with being male and with engaging in unhealthy lifestyle behaviors such as not having breakfast, poor sleep quality, and some problematic Internet usage. Although the prevalence of unhappiness was significantly higher among current smokers and alcohol drinkers, these behaviors were not associated with unhappiness in the multivariable logistic regression analysis. Unhappiness among Japanese adolescents appears to be strongly related to how they spend their daily life. We therefore consider it desirable for school officials to educate students on the importance of happiness and lifestyle factors conducive to happiness.

## Introduction

Subjective happiness has received increasing attention from academics and policymakers around the world as a global measure of subjective well-being [1, 2]. In the psychometric literature, subjective well-being consists of evaluative and affective elements [3]; evaluative well-being reflects individuals’ judgments about the quality and virtue of their lives [4], while affective well-being reflects positive emotions and moods such as happiness, joy, excitement, and cheerfulness, as well as avoidance of pain and depression [5]. Although there are many examples of people who have achieved meaning and fulfillment in life without enjoyment, the different types of subjective well-being are on average positively correlated [6]. Thus, many regard subjective happiness as a major life goal [1] with numerous positive effects on physical and mental health, including improved cardiovascular function [7], improved sleep quality [8], an increased survival rate [9], and increased quality of life [10].

However, the above findings pertain to adults only; few studies have targeted adolescents. Adolescence is a period of rapid physical and psychological change intrinsically linked to adulthood [8]—indeed, happiness in adolescence is associated with happiness in adulthood. Furthermore, happiness in childhood is related to social and coping skills (which predict subjective happiness) in adulthood [11]. Happiness in adolescence is also associated with lifestyle behaviors such as physical activity, eating habits, smoking, and alcohol use [12–14]. For instance, a cross-sectional survey in Hong Kong targeted 45,857 secondary school students and found that students who reported fewer than two days of exercise per week were up to 32% more likely to be unhappy than those who reported three to seven days of exercise. Further, the risk of unhappiness increased with the number of days of drinking, and current and former smokers were more likely to be unhappy than those who never smoked [13]. Subjective happiness is also negatively related to problematic Internet usage [15–17]. A cross-sectional study of 56,086 adolescents in Taiwan showed that adolescents who reported being unhappy had a higher risk of Internet addiction—1.54 times higher in boys and 1.88 times higher in girls—than adolescents who were happy [16]. A study in Chile targeting 3461 students 17 to 24 years old reported that eating lunch, fruits, and vegetables every day increased the likelihood of being classified as "very happy" [14]. Thus, happy adolescents are less likely to engage in a variety of harmful and unhealthy behaviors, including smoking, drinking, unhealthy eating, and problematic Internet usage. Increasing adolescents’ subjective happiness might improve their social adaptability and quality of life later on [18, 19]. Therefore, the positive impacts of subjective happiness can benefit health through indirect relationships with health promotion activities.

A study targeting 540,000 fifteen-year old students found that the happiness levels of Japanese students was ranked 42 out of 47 countries and regions (including Organisation for Economic Co-operation and Development [OECD] countries and partner nations) [20]. As such, it is important to implement strategies aimed at improving Japanese youths’ overall happiness, which requires consideration of the factors related to happiness, such as gender [14] and environmental factors, including school support structures, communication between parents and children, and academic load [21]. Teachers and policymakers would find it challenging to improve parent-child relationships as a method of increasing adolescents’ happiness. Though change is not an immediate process, addressing lifestyle behaviors and the school environment while attempting to address parent-child relationship quality might result in more immediate benefits than solely focusing on a single factor.

No studies have actually investigated the associations between lifestyle factors and unhappiness in Japanese adolescents. In 2000, the Government of Japan formulated a comprehensive health policy, Health Japan21, and set daily lifestyle goals such as increasing breakfast intake and reducing drinking and smoking in minors [22]. Internet addiction has also become a serious public health issue, and its effects are particularly detrimental to young people, who are in the process of growing both mentally and socially [23]. Targeting lifestyle factors allows us to orient adolescents toward future happiness by improving the conditions of not-yet-happy adolescents. Although there have been previous reports on “unhappiness at school” in a nationwide survey [24], we were interested in the “feeling that school is not fun” (based on the Japanese wording of the questionnaires) rather than unhappiness. In actuality, numerous extracurricular activities and aspects of adolescent lifestyle occur outside of school; therefore, increasing the range of variables is needed, including not only school life, but also daily life behaviors to accurately evaluate unhappiness in adolescents. Identifying the factors associated with subjective unhappiness among adolescents is a step toward addressing unhappiness in adolescents and thereby improving individuals’ future physical and mental health. Therefore, we conducted a large-scale survey on how different lifestyle factors among adolescents throughout Japan are associated with unhappiness. Based on previous research, we hypothesized that adolescents’ levels of unhappiness would be associated with daily behaviors, such as Internet usage; whether they participate in school extracurricular activities, sleep quality, eating behavior; and gender. Daily activities facilitate group intervention education in schools, and considering which behaviors are associated with adolescents can inform future surveys and policies on lifestyle interventions to improve happiness.

## Materials and methods

### Participants

We collected data via the 2017–2018 Lifestyle Survey of Adolescents. Of the 10,235 junior and 4,907 senior high schools registered in Japan in May 2017, we sampled 98 junior (selection rate: 0.96%) and 86 senior (selection rate: 1.75%) high schools using a stratified, single-stage cluster-sampling method, which involved dividing Japan into regional blocks and randomly selecting schools from each block. We used this method to limit sampling bias. All students in the sampled schools were included. The sample size was determined using the response rates and confidence intervals based on variance of results obtained from a previous study [25]. Replies were obtained from 48 junior high schools (school response rate: 49.0%) and 55 senior high schools (school response rate: 64.0%; total school response rate: 56.0%) and enrolled 118,303 students in the study. A total of 64,417 individuals responded. We excluded 88 questionnaires lacking gender information or with inconsistent responses. Consequently, 64,329 questionnaires were analyzed (effective response rate = 54.4%). The age range was 12–19 years (mean = 15.7±1.7 years).

### Survey procedure

We sent a letter to the principals of selected schools requesting their cooperation, along with questionnaire forms and envelopes for enrolled students. In participating schools, we had class teachers inform students of the study, including its confidentiality and voluntary participation, and assure them that their privacy would be protected. There was an option to withdraw from the study or refuse to complete the questionnaire. Teachers delivered the completed questionnaires in the sealed envelopes back to our office. The survey was administered between December 2017 and February 2018.

### Measures

The questionnaire assessed (1) personal data, (2) lifestyle behaviors, (3) school life, and (4) subjective happiness. Personal data included school type (junior high school or senior high school), gender, age, and school grade.

For lifestyle behaviors, we assessed frequency of having breakfast (“every day,” “sometimes,” or “seldom”), drinking and smoking status, subjective sleep quality (“very good,” “good,” “bad,” or “very bad), and Internet usage. As for eating breakfast, respondents who selected “every day” were included in the “yes” category and those who selected “sometimes” or “never” were collapsed into the “no” category for analysis [26]. Participants were defined current smokers if they answered that they had smoked one cigarette per day or more in the past month. Similarly, we defined those who responded that they had drunk alcohol one day or more in the past month as being current drinkers [26]. For subjective sleep quality, participants who responded with “bad” or “very bad” were considered to have poor subjective sleep quality [26]. To assess Internet usage, we used the 8-item version of the Young Diagnostic Questionnaire for Internet Addiction (YDQ) [27]. Each item is rated dichotomously (“yes” or “no”).

As for school life, participation in extracurricular activities (“active participation,” “passive participation,” or “no participation”) and future direction were asked. To evaluate future plan, we used the following question. “What is your plan for your future life course?” Participants selected one of seven items: “high school,” “vocational school,” “college,” “university,” “postgraduate school,” “taking a job after leaving the current school,” and “not decided yet.” Those who selected “university” or “postgraduate school” were grouped as students who intended to go to university; otherwise, they were grouped as students who did not intend to go to university or those who had not yet decided [26].

Subjective happiness was measured using a single item: “In general, how would you describe your happiness?” Participants responded on a visual analogue scale. Participants were instructed to (a) focus on their global estimation and general feelings, (b) take note that 0 is the minimum and 10 is the maximum, and (c) select the number that best described their feelings. This single scale has good concurrent, convergent, and divergent validity [28]. Single-item happiness measures have been used widely throughout the world [29, 30]. We defined participants with a score of 3 or less (10th percentile score) as being unhappy [31], because it is quite plausible that data on lower levels of happiness scores (that is, unhappiness) could help to identify groups or problems that are potential priorities for policy interventions. Thus, subjective unhappiness was dichotomously scored (unhappy = 1, other = 0).

### Data analysis

First, we examined participants’ characteristics by school type. Second, we calculated the prevalence and 95% confidence intervals (95% CI) of unhappiness by school grade. Third, we examined the prevalence of unhappiness by lifestyle behaviors and items of Young Diagnostic Questionnaire for Internet Addiction, according to gender and school type, by using the χ^2^ test. Finally, we analyzed a multivariable logistic regression, calculating the adjusted odds ratios (ORs) of each factor and its 95% CI for subjective unhappiness. The covariates in the logistic regression analysis included basic demographic characteristics (gender and school grade), lifestyle behaviors (having breakfast, drinking alcohol, smoking status, sleep quality, and Internet usage), and school life (extracurricular activities and intending to study at university). To determine covariates, we referred to factors associated with happiness in previous studies [14, 15, 17, 24]; these behaviors are factors of high interest to national policies as well as teachers and parents as intervention goals. The statistical level of significance was p<0.01. Statistical analyses were performed with Stata 15.1.

### Ethics statement

In the Ethical Guidelines for Epidemiological Studies jointly announced by the Ministry of Health, Labour and Welfare and the Ministry of Education, Culture, Sports, Science and Technology of Japan, personal information is defined as follows: information of a living individual, and the name, birthday, and other descriptions included in that information that can be used to identify a specific individual. We used questionnaires devoid of all such information to prevent participant identification and to safeguard their privacy. We obtained written informed consent from all participants. Limited to junior high school students, informed consent was obtained from each child's parent or guardian. According to the Ministry of Health, Labor and Welfare's epidemiology ethical guidelines, researchers are not required to obtain parental approval in a non-invasive survey of high school students. This study was approved by the ethical review board of the Tottori University School of Medicine.

## Results

Table 1 shows the participant demographics by school type. The prevalence of subjective unhappiness was 9.3% and 10.2% in junior and senior high school students, respectively. Students in junior and senior high schools who wished to go to university accounted for 61.2% and 55.5%, while 65.3% and 51.8% actively participated in extracurricular activities, respectively. Most junior and senior high school students ate breakfast daily (85.9% and 81.5%, respectively), while 29.5% and 39.7% had poor sleep quality. About half of adolescents reported being preoccupied with the Internet and regularly stayed online longer than they originally intended.

**Table 1:** Characteristics of the analyzed participants.

		Junior high school		Senior high school	
		n	%	n	%
Gender					
	Boys	11,179	50.3	23,403	55.8
	Girls	11,036	49.7	18,534	44.2
Grade					
	Grade 7 and 10	7,384	33.4	14,201	34.0
	Grade 8 and 11	7,329	33.1	14,212	34.0
	Grade 9 and 12	7,415	33.5	13,404	32.0
Subjective happiness					
	Unhappy	2,057	9.3	4,264	10.2
	Neither	7,558	34.0	16,718	39.9
	Happy	11,872	53.4	19,680	46.9
	Unknown	728	3.3	1,275	3.0
Having breakfast					
	Every day	19,079	85.9	34,183	81.5
	Sometimes	1,627	7.3	4,052	9.7
	Seldom	887	4.0	2,603	6.2
	Unknown	622	2.8	1,099	2.6
Participating in extracurricular activities					
	No	3,896	17.5	13,227	31.6
	Active	14,502	65.3	21,740	51.8
	Passive	3,103	14.0	5,796	13.8
	Unknown	714	3.2	1,174	2.8
Present smoking					
	No	21,971	98.9	40,998	97.8
	Yes	244	1.1	939	2.2
Present alcohol use					
	No	21,580	97.1	38,986	93.0
	Yes	635	2.9	2,951	7.0
Subjective sleep quality					
	Good	14,937	67.2	24,063	57.4
	Bad	6,559	29.5	16,633	39.7
	Unknown	719	3.3	1,241	2.9
Intending to study at university					
	Yes	13,587	61.2	23,262	55.5
	No	4,253	19.1	14,241	34.0
	Not yet decided	3,704	16.7	3,322	7.9
	Unknown	671	3.0	1,112	2.7
Internet Addiction Diagnostic Questionnaire					
	Preoccupied with the Internet	10,981	49.7	23,359	56.0
	Need to use the Internet with increasing amounts of time to achieve satisfaction	2,430	11.0	5,109	12.2
	Repeatedly made unsuccessful efforts to control, cut back, or stop Internet use	8,169	37.0	16,232	38.9
	Restless, moody, depressed, or irritable when attempting to cut down or stop Internet use	4,382	19.8	8,509	20.4
	Stay online longer than originally intended	9,196	41.6	22,999	55.1
	Jeopardized or risked the loss of significant relationship, school, educational or club activity because of the Internet	1,586	7.2	3,871	9.3
	Lied to family members, therapist, or others to conceal the extent of involvement with the Internet	3,212	14.5	6,900	16.5
	Use the Internet as a way of escaping from problems or of relieving a dysphoric mood	4,003	18.1	11,658	27.9

Internet Addiction Diagnostic Questionnaire by Young’s Diagnostic Questionnaire showed that participants answered "yes".

Table 2 shows the prevalence of unhappiness by school grade. The average prevalence was 10.2%. The prevalence increased with higher grade, while has leveled off since Grade 10.

**Table 2:** Prevalence of subjective unhappiness by school grade.

	Total number	Prevalence of subjective unhappiness (%)	95% CI		p-value
Grade 7	7,384	9.0	8.3	9.7	0.003
Grade 8	7,329	9.5	8.9	10.2	(χ^2^(5) = 17.8)
Grade 9	7,415	10.1	9.4	10.8	
Grade 10	14,201	10.5	10.0	11.0	
Grade 11	14,212	10.5	10.0	11.0	
Grade 12	13,404	10.5	9.9	11.0	
Total	63,945	10.2	9.9	10.4	

Participants with missing data were excluded from the analysis.

P-value was calculated with chi-square test by grade.

Unhappiness was defined as a score of 3 or less on an 11-point scale.

**Fig 1:**
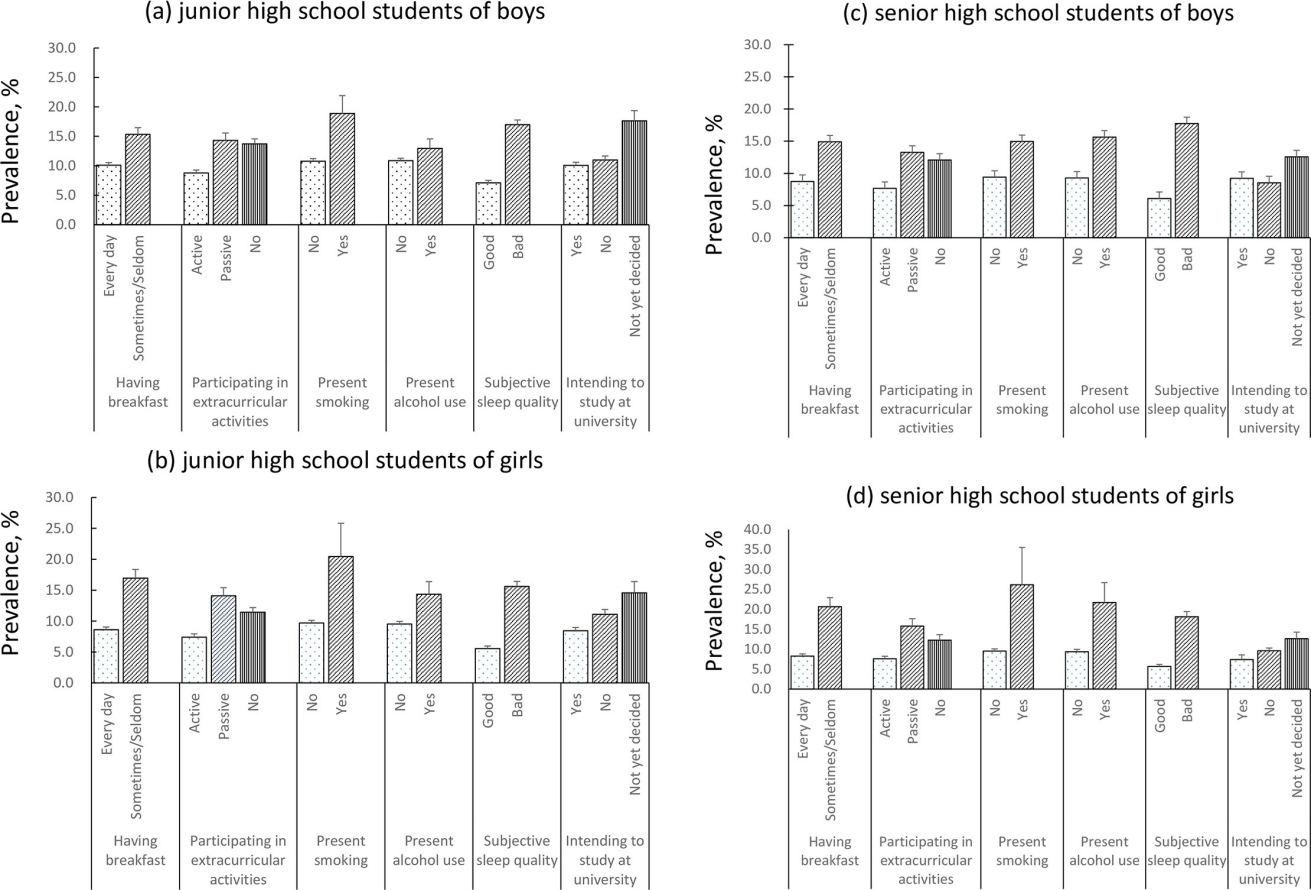
The prevalence of subjective unhappiness in Japanese adolescents by each life style factor. (a) junior high school students of boys (b) junior high school students of girls (c) senior high school students of boys (d) senior high school students of girls.

Fig 1 shows the prevalence of subjective unhappiness in lifestyle behaviors and school life by each school type and gender. Among junior and high school students, factors that had significantly a higher prevalence of subjective unhappiness were having breakfast sometimes/seldom, passive participation in extracurricular activities, present smoking, present alcohol use, poor sleep quality, and not yet having decided to study at university. These variables showed no significant differences by gender among both school levels.

Tables 3 and 4 show the prevalence of subjective unhappiness in items by each school type and gender. In all YDQ items, the prevalence of unhappiness among those who answered “Yes” was higher than that among those who answered “No.” In particular, items with a large difference in the prevalence of unhappiness were “Need to use the Internet with increasing amounts of time to achieve satisfaction,” “Restless, moody, depressed, or irritable when attempting to cut down or stop Internet use,” “Jeopardized or risked the loss of significant relationship, school, educational or club activity because of the Internet,” and “Use the Internet as a way of escaping from problems or of relieving a dysphoric mood.”

**Table 3:** The prevalence of subjective unhappiness in junior high school students by components of internet addiction.

	Boys						Girls					
	n	%	95% CI			p-value	n	%	95% CI			p-value
Preoccupied with the Internet												
No	417	7.9	7.2	-	8.7	< 0.001	389	7.1	6.5	-	7.9	< 0.001
Yes	585	11.0	10.2	-	11.9		654	12.3	11.4	-	13.2	
Need to use the Internet with increasing amounts of time to achieve satisfaction												
No	772	8.3	7.7	-	8.8	< 0.001	800	8.2	7.6	-	8.7	< 0.001
Yes	232	18.0	16.0	-	20.3		247	23.7	21.1	-	26.4	
Repeatedly made unsuccessful efforts to control, cut back, or stop Internet use												
No	603	8.6	8.0	-	9.3	< 0.001	537	8.3	7.7	-	9.0	< 0.001
Yes	398	11.1	10.1	-	12.1		507	11.7	10.8	-	12.7	
Restless, moody, depressed, or irritable when attempting to cut down or stop Internet use												
No	665	7.7	7.1	-	8.3	< 0.001	656	7.7	7.1	-	8.2	< 0.001
Yes	336	17.2	15.6	-	19.0		393	17.2	15.7	-	18.9	
Stay online longer than originally intended												
No	530	8.2	7.5	-	8.9	< 0.001	463	7.7	7.1	-	8.4	< 0.001
Yes	471	11.4	10.5	-	12.4		579	12.1	11.2	-	13.1	
Jeopardized or risked the loss of significant relationship, school, educational or club activity because of the Internet												
No	861	8.7	8.2	-	9.3	< 0.001	887	8.9	8.3	-	9.4	< 0.001
Yes	136	19.6	16.7	-	22.7		162	19.4	16.8	-	22.2	
Have you lied to family members, therapist, or others to conceal the extent of involvement with the Internet												
No	783	8.5	7.9	-	9.1	< 0.001	763	8.4	7.8	-	9.0	< 0.001
Yes	219	15.9	14.0	-	17.9		287	16.7	15.0	-	18.5	
Use the Internet as a way of escaping from problems or of relieving a dysphoric mood												
No	667	7.3	6.8	-	7.9	< 0.001	537	6.4	5.9	-	7.0	< 0.001
Yes	334	22.9	20.7	-	25.1		508	21.1	19.4	-	22.8	

Participants with missing data were excluded from the analysis. P-values were calculated by chi-square test for all factors.

**Table 4:** The prevalence of subjective unhappiness in senior high school students by components of internet addiction.

	Boys						Girls					
	n	%	95% CI			p-value	n	%	95% CI			p-value
Preoccupied with the Internet												
No	979	9.6	9.0	-	10.2	< 0.001	571	7.5	7.0	-	8.2	< 0.001
Yes	1,485	12.2	11.6	-	12.8		1,212	11.5	10.9	-	12.1	
Need to use the Internet with increasing amounts of time to achieve satisfaction												
No	1,898	9.8	9.3	-	10.2	< 0.001	1,379	8.5	8.1	-	9.0	< 0.001
Yes	573	19.0	17.7	-	20.5		401	20.7	18.9	-	22.6	
Repeatedly made unsuccessful efforts to control, cut back, or stop Internet use												
No	1,495	10.1	9.6	-	10.6	< 0.001	886	8.9	8.4	-	9.5	< 0.001
Yes	977	12.9	12.1	-	13.6		894	10.9	10.2	-	11.6	
Restless, moody, depressed, or irritable when attempting to cut down or stop Internet use												
No	1,732	9.4	9.0	-	9.9	< 0.001	1,120	8.1	7.6	-	8.5	< 0.001
Yes	728	18.4	17.2	-	19.6		676	15.6	14.4	-	16.6	
Stay online longer than originally intended												
No	1,093	9.8	9.2	-	10.3	< 0.001	596	8.5	7.8	-	9.1	< 0.001
Yes	1,369	12.2	11.6	-	12.9		1,192	10.7	10.1	-	11.3	
Jeopardized or risked the loss of significant relationship, school, educational or club activity because of the Internet												
No	2,065	10.1	9.7	-	10.5	< 0.001	1,421	8.7	8.3	-	9.2	< 0.001
Yes	401	20.7	18.9	-	22.6		360	19.8	18.0	-	21.7	
Have you lied to family members, therapist, or others to conceal the extent of involvement with the Internet												
No	1,855	9.8	9.4	-	10.2	< 0.001	1,312	8.8	8.3	-	9.3	< 0.001
Yes	614	17.8	16.5	-	19.1		473	14.6	13.4	-	15.9	
Use the Internet as a way of escaping from problems or of relieving a dysphoric mood												
No	1,434	8.2	7.8	-	8.6	< 0.001	690	5.9	5.5	-	6.3	< 0.001
Yes	1,022	20.9	19.8	-	22.1		1,099	17.0	16.1	-	18.0	

Participants with missing data were excluded from the analysis. P-values were calculated by chi-square test for all factors.

Table 5 shows the crude and adjusted ORs (AORs) and 95% CIs for the association between unhappiness and each of the explanatory variables. In this multivariable logistic regression model, the AUC was 0.724 and the pseudo-R squared was 0.093. The analysis revealed higher odds of unhappiness among individuals with poor sleep quality (AOR 2.41; 95% CI 2.28–2.55); those with passive participation in extracurricular activities (AOR 1.12; 95% CI 1.04–1.22); those who used the Internet as a way of escaping from problems or of relieving a dysphoric mood (AOR 2.44; 95% CI 2.29–2.61)); those with the need to use the Internet increasingly to achieve satisfaction (AOR 1.55; 95% CI 1.43–1.67); those who were restless, moody, depressed, or irritable when attempting to cut down or stop Internet use (AOR 1.32; 95% CI 1.23–1.42); and those who had jeopardized or risked the loss of significant relationships or school, educational, or club activities because of the Internet (AOR 1.35; 95% CI 1.24–1.47). Conversely, lower odds of unhappiness were found for girls (AOR 0.80; 95% CI 0.71–0.79), individuals who ate breakfast daily (AOR 0.75; 95% CI 0.70–0.81), individuals intending to study at university (AOR 0.87; 95% CI 0.73–0.83), and, interestingly, those preoccupied with the Internet (AOR 0.88; 95% CI 0.82–0.93), those who repeatedly made unsuccessful efforts to control, cut back, or stop Internet use (AOR 0.88; 95% CI 0.83–0.94), and those who stayed online longer than originally intended (AOR 0.85; 95% CI 0.79–0.90). School grades, smoking, and drinking status were not significant related factors.

**Table 5:** Logistic regression results: Variables associating unhappiness in Japanese adolescents.

		N	Crude OR	95%CI		p-value	AOR	p-value		
Gender										
	Boys	32,119	1.00				1.00			
	Girls	28,276	0.92	0.87	0.97	0.002	0.80	0.76	0.85	<0.001
Grade										
	Grade 7	6,814	1.00							
	Grade 8	6,893	1.07	0.95	1.19	0.267	0.98	0.87	1.11	0.801
	Grade 9	6,997	1.14	1.02	1.27	0.023	0.97	0.86	1.09	0.612
	Grade 10	13,496	1.18	1.07	1.30	0.001	0.95	0.86	1.06	0.393
	Grade 11	13,458	1.18	1.07	1.30	0.001	0.92	0.83	1.03	0.140
	Grade 12	12,737	1.18	1.07	1.31	0.001	0.90	0.81	1.01	0.081
Having breakfast										
	Sometimes/seldom	8,808	1.00				1.00			
	Everyday	51,587	0.51	0.48	0.54	<0.001	0.75	0.70	0.81	<0.001
Participating in extracurricular activities										
	No	17,036	1.00				1.00			
	Active	36,061	0.61	0.57	0.64	<0.001	0.76	0.71	0.81	<0.001
	Passive	8,862	1.20	1.12	1.29	<0.001	1.12	1.04	1.22	0.005
Present smoking										
	No	59,366	1.00				1.00			
	Yes	1,029	2.15	1.85	2.51	<0.001	1.21	1.01	1.46	0.038
Present drinking										
	No	57,120	1.00				1.00			
	Yes	3,275	1.52	1.38	1.68	<0.001	0.99	0.88	1.11	0.890
Subjective sleep quality										
	good	37,831	1.00				1.00			
	bad	22,564	3.05	2.89	3.22	<0.001	2.41	2.28	2.55	<0.001
Intending to study at university										
	No	27,647	1.00				1.00			
	Yes	27,452	0.90	0.85	0.95	<0.001	0.87	0.82	0.93	<0.001
	Not yet decided	6,967	1.49	1.38	1.61	<0.001	1.30	1.19	1.41	<0.001
Internet Addiction Diagnostic Questionnaire										
	Preoccupied with the Internet	34,340	1.01	0.94	1.07	0.881	0.88	0.82	0.93	<0.001
	Need to use the Internet with increasing amounts of time to achieve satisfaction	7,539	1.01	0.95	1.08	0.746	1.55	1.43	1.67	<0.001
	Repeatedly made unsuccessful efforts to control, cut back, or stop Internet use	24,401	0.75	0.70	0.81	<0.001	0.88	0.83	0.94	<0.001
	Restless, moody, depressed, or irritable when attempting to cut down or stop Internet use	12,891	1.65	1.52	1.78	<0.001	1.32	1.23	1.42	<0.001
	Stay online longer than originally intended	32,195	1.08	1.02	1.15	0.013	0.85	0.79	0.90	<0.001
	Jeopardized or risked the loss of significant relationship, school, educational or club activity because of the Internet	5,457	1.22	1.15	1.29	<0.001	1.35	1.24	1.47	<0.001
	Lied to family members, therapist, or others to conceal the extent of involvement with the Internet	10,112	0.95	0.89	1.02	0.185	1.06	0.98	1.14	0.119
	Use the Internet as a way of escaping from problems or of relieving a dysphoric mood	15,661	1.50	1.38	1.63	<0.001	2.44	2.29	2.61	<0.001

Abbreviations: AOR = adjusted odds ratio, CI = confidence interval.

Unhappiness: Students who selected from 0 to 3 point scales about happiness.

Participants for whom data were missing were excluded from the analyses.

All the items included in this table were input as covariates in this multivariable logistic model.

## Discussion

This is the first nationally representative study to examine the association between subjective unhappiness and daily lifestyle factors such as having breakfast, extracurricular activity, sleep, and Internet usage in Japanese adolescents. The results demonstrated that unhappiness was significantly positively associated with passive participation or lack of participation in extracurricular activities, poor sleep quality, and some aspects of problematic Internet usage. On the other hand, unhappiness was significantly negatively associated with being a girl, eating breakfast daily, intending to study at university, and a few problematic Internet usage patterns. The results were nearly identical with previous research in other countries and confirmed our hypothesis except the lack of effects on class grades. These findings provide important evidence on the ways that lifestyle interventions could improve overall happiness in adolescents in Japan.

We found that there were unhappier boys than girls in Japan. Gender differences in subjective happiness and well-being have been reported in both Western and Asian countries [14]. Male Chinese adolescents had 1.05-fold greater odds of being unhappy than did their female counterparts [13]. This difference might be attributed to women having higher resilience than men [32]. However, an Australian study reported that no gender differences in subjective well-being were found among high school students [33]. Our present study has unequal ratios between male and female samples and should be standardized in future studies. Thus, the gender difference in adolescent happiness remains unclear. Adolescence is a period of profound change (e.g., hormonal balance, cultural and social influences), which future studies should examine. Liu also reported that, as boys get older, their happiness falls significantly; girls, however, are unaffected [34], which coincides with our findings. No prior studies were available to investigate whether this difference lasts into adulthood. Longitudinal research is needed to examine changes in subjective happiness over time in males and females.

Our raw data showed that students’ grade in school was associated with unhappiness. However, our multivariate logistic regression analysis found no association between grade level and unhappiness. Research on adolescents in China [13], Chile [14], and Europe/North America [35] reported that the risk of unhappiness increased with age. Thus, we predicted that as students move up in grade, their proneness to unhappiness would increase (due to the added burden of cram schools, after-school activities, longer commuting hours, and less free time). One reason for the discrepancy in our results could be that some of the explanatory variables were confounders between class grades. A second reason may be related to the Japanese education system, which has students prepare for academic entrance exams in grades 9 and 12. Thus, students are under constant academic pressure. Furthermore, even when the academic pressure lessens, students engage in extracurricular activities; thus, they have little free time regardless.

Not eating breakfast regularly was associated with an increased risk of unhappiness. A cross-sectional study of Chilean university students similarly revealed that happiness was positively associated with regularly eating breakfast [14]. Possibly, breakfast consumption helps maintain normal weight status in adolescents [36]. Given that obesity has a negative effect on subjective happiness [37], eating breakfast might be related to subjective happiness via weight management.

Surprisingly, a passive attitude toward extracurricular activities had a stronger correlation with unhappiness than did no participation at all. Extracurricular activities often involve interaction with individuals with similar objectives and interests [38]. Students with passive attitudes might be unable to commit to these objectives and interests, thus making them feel more isolated within a group. Most Japanese junior and senior high school students are obligated to participate in extracurricular activities. They are often extremely busy with extracurricular activities, which typically occur from early morning until late at night, and frequently on weekends. Therefore, students are unlikely to feel happy if they are not interested in these activities. Given this result, educators need to change the way students participate in extracurricular activities.

The hope to study at university in the future was associated with a decreased risk of unhappiness. We might attribute to this to the fact that individuals who advance to university-level education set goals for themselves, achieve relatively good grades [39], or have a positive home environment [11], all of which may make them more unlikely to become unhappy.

These two factors (participation in extracurricular activities and intend to study at university) seem to be related to freedom of choice. Those who are passively participating are supposed to escape from their activities. However, they are considered to be in a situation where they are forced to participate because they are bound by the relationships between their neighbors and the rules of the school. Therefore, students who participate passively in extracurricular activities have a higher odds ratio of unhappiness than students who do not participate. In addition, it can be seen that the reason for not intending to study at university is that the options for the future are narrowed, such as having to work due to economic circumstances or having no interest in study. Just as discretion is one of the key work engagement factors for workers [40], freedom of choice may be a related factor of happiness. Future research requires a longitudinal study of how self-determination is related to happiness.

Interestingly enough, the multivariable logistic regression results showed there were no statistical associations of drinking alcohol and smoking with unhappiness. Both negative correlations between smoking and happiness [13, 41] and no correlation [14, 42] have been found in previous longitudinal studies. Thus, we cannot conclude definitively whether a correlation exists or not. Similarly, there are no consistent findings on the correlation between drinking alcohol and happiness [13, 14, 42]. A notable difference between this study and past ones is that the prevalence of smoking and drinking among Japanese junior and senior high school students is decreasing [43, 44]; currently, these prevalence rates are extremely low, and the population of drinkers was not stratified in this study. Furthermore, in Japan, it is illegal for people below 20 years of age to smoke and drink alcohol; therefore, students often refuse to respond to questions on smoking and drinking.

In particular, poor sleep quality was associated with higher risk for unhappiness. A longitudinal study of adults found a J-shaped relationship between happiness and sleep duration [45], indicating that subjective unhappiness decreased with sleep duration. This finding suggested that getting enough sleep was associated with increased happiness. However, few studies have examined the correlation between sleep and happiness among adolescents. Roberts et al. reported that those with insomnia have 3.27-fold higher odds of identifying themselves as unhappy compared to those who do not have insomnia [46]. In contrast, a cross-sectional study of 750 Americans aged 14–15 years revealed that happiness had a significant correlation with daily activities such as studying, communicating with friends on screens, and spending time with family, but not with sleep deprivation [47]. Further research on the relationship between sleeping disorders and happiness is required.

In line with previous research [15–17], we thought that all Internet-dependent items would be positively related factors of unhappiness in adolescence. Surprisingly, our findings showed that a few components of Internet addiction were negatively correlated with unhappiness. These results may suggest that the Internet brings both happiness and unhappiness to adolescents. Similarly, a European pooled cross-sectional dataset showed that Internet usage has been found to correlate positively with well-being [48]. A study of Chinese adolescents revealed that excessive Internet usage provides temporary enjoyment, but ultimately suppresses long-term well-being [17]. A nationally representative yearly survey in the United States showed that American adolescents found that limiting time spent in electronic communication is associated with the greatest happiness levels [49].

Our data did show, however, that the destruction of relationships through Internet use was associated with unhappiness. SNSs like Facebook can generate negative feelings (e.g., jealousy) [50]; constant access to friends’ profiles provides people with more in-depth information on others than ever before, but it may cause jealousy and decrease subjective happiness if individuals see friends interacting with ideal figures or role models. Our data also showed that Internet use for the purpose of escape and feeling anxious or depressed by using Internet were associated with unhappiness. Indeed, subjective happiness might be reduced by social isolation, as real relationships become diluted through prolonged Internet use. Adolescents are prone to problematic Internet usage because of their underdeveloped emotional regulation and self-control [51]. Internet addiction does not help adolescents establish interpersonal relationships in the real world. Therefore, given that we live in an Internet-connected world, schools and families need to instruct students on methods of proper Internet usage to maintain adolescents’ happiness.

This study has several limitations. First, because this was a cross-sectional study, we cannot determine the causal relationships for each factor, or how these might change over time. Thus, future studies should use a longitudinal design. Second, we could not collect data from students who were absent from school on the survey day as well as school dropouts. The prevalence of unhappiness might be even higher among absentees and dropouts when compared to those in attendance on the day of the study. Similarly, the data of the explanatory variables might have been affected by the exclusion of these individuals. Third, no data were obtained on participants’ weight or socioeconomic factors, such as family income or parents’ educational levels. Some studies have highlighted the importance of socioeconomic status for subjective happiness [52], whereas others have found no relation between social class and subjective happiness [53]. Thus, future research should include socioeconomic factors. Fourth, the response rate in this study was 56.0%; thus, approximately 44% of the students did not participate. There may be two reasons for the non-responsiveness. The first reason is that people below 20 years old in Japan are prohibited by law from smoking and drinking alcohol. Therefore, schools and students tend to be non-cooperative in responding to a survey that includes questions on smoking and drinking alcohol. The second reason is the epidemiological survey in Japan required the consent of parents in junior high school students, which can be difficult to obtain. Finally, in this study the effect size, an essential component when evaluating the strength of a statistical claim, was rather small. However, a small effect size can be of great practical value. This is especially true if a treatment is relatively cheap, easy to perform, politically viable, and can be used on a large scale, thereby affecting a large number of individuals [54]. In addition to that practical value, this study had three main strengths: 1) it is a nationwide survey; 2) it has an extremely large sample; and 3) it has a survey response rate over 50%, which was high for this type of epidemiological study. These strengths provide increased credibility in our results by minimizing the impact of potential random errors from self-reporting, especially among adolescents.

## Conclusions

This large-scale Japanese adolescent study showed that unhappiness was strongly associated with being male, engaging in unhealthy lifestyle behaviors (e.g., not having breakfast, poor sleep quality), and worrisome Internet usage patterns. Given that our study showed that passive participants in extracurricular activities were less happy, education officials should consider whether to require such activities. Teachers and parents should emphasize the importance of sleep and ensure that adolescents are able to get the sleep they need. Further, schools and families should instruct students in how to use the Internet and provide limits to Internet use. Our findings can help inform future government policies and help teachers and parents promote good quality of life among adolescents.
